# A combined cell-consortium approach for lignocellulose degradation by specialized *Lactobacillus plantarum* cells

**DOI:** 10.1186/1754-6834-7-112

**Published:** 2014-07-24

**Authors:** Sarah Moraïs, Naama Shterzer, Raphael Lamed, Edward A Bayer, Itzhak Mizrahi

**Affiliations:** Department of Biological Chemistry, The Weizmann Institute of Science, 234 Herzl St, Rehovot, 7610001 Israel; Department of Ruminant Science, Institute of Animal Sciences, Agricultural Research Organization, P.O.B. 6, Bet-Dagan, 50250 Israel; Department of Molecular Microbiology and Biotechnology, Tel Aviv University, P.O. Box 39040, Ramat Aviv, 69978 Israel

**Keywords:** Designer cellulosome, Biomass, Bioprocessing, Wheat straw, Glycoside hydrolase, Enzymatic paradigm, Spatial differentiation, Biomimicry

## Abstract

**Background:**

*Lactobacillus plantarum* is an attractive candidate for metabolic engineering towards bioprocessing of lignocellulosic biomass to ethanol or polylactic acid, as its natural characteristics include high ethanol and acid tolerance and the ability to metabolize the two major polysaccharide constituents of lignocellulolytic biomass (pentoses and hexoses). We recently engineered *L. plantarum* via separate introduction of a potent cellulase and xylanase, thereby creating two different *L. plantarum* strains. We used these strains as a combined cell-consortium for synergistic degradation of cellulosic biomass.

**Results:**

To optimize enzymatic degradation, we applied the cell-consortium approach to assess the significance of enzyme localization by comparing three enzymatic paradigms prevalent in nature: (i) a secreted enzymes system, (ii) enzymes anchored to the bacterial cell surface and (iii) enzymes integrated into cellulosome complexes. The construction of the three paradigmatic systems involved the division of the production and organization of the enzymes and scaffold proteins into different strains of *L. plantarum*. The spatial differentiation of the components of the enzymatic systems alleviated the load on the cell machinery of the different bacterial strains. Active designer cellulosomes containing a xylanase and a cellulase were thus assembled on *L. plantarum* cells by co-culturing three distinct engineered strains of the bacterium: two helper strains for enzyme secretion and one producing only the anchored scaffoldin. Alternatively, the two enzymes were anchored separately to the cell wall. The secreted enzyme consortium appeared to have a slight advantage over the designer cellulosome system in degrading the hypochlorite pretreated wheat straw substrate, and both exhibited significantly higher levels of activity compared to the anchored enzyme consortium. However, the secreted enzymes appeared to be less stable than the enzymes integrated into designer cellulosomes, suggesting an advantage of the latter over longer time periods.

**Conclusions:**

By developing the potential of *L. plantarum* to express lignocellulolytic enzymes and to control their functional combination and stoichiometry on the cell wall, this study provides a step forward towards optimal biomass bioprocessing and soluble fermentable sugar production. Future expansion of the preferred secreted-enzyme and designer-cellulosome systems to include additional types of enzymes will promote enhanced deconstruction of cellulosic feedstocks.

**Electronic supplementary material:**

The online version of this article (doi:10.1186/1754-6834-7-112) contains supplementary material, which is available to authorized users.

## Background

In nature, the degradation of the plant cell wall is carried out by a variety of different cellulolytic microorganisms, by using cellulases and associated carbohydrate-active enzymes, such as xylanases and other glycoside hydrolases, carbohydrate esterases and polysaccharide lyases [1]. These enzymes are employed in various recognized paradigms [2]. In this context, the various enzymes may be ‘freely’ secreted, as abundant in aerobic fungi and bacteria [3]. Alternatively, the enzymes may be anchored to the cell surface [4]. In addition, the complement of enzymes may be integrated into highly efficient complexes called cellulosomes (produced by anaerobic bacteria) that are composed of numerous functional protein modules which interact with each other (via cohesin-dockerin interactions) and with the substrate (via carbohydrate-binding modules), in order to synergistically degrade lignocellulosic biomass.

These enzymatic paradigms have individually been the subject of extensive research and engineering to augment the action of natural systems in the intricate degradation of plant cell walls [5, 6]. Numerous attempts in transforming bacterial cells and fungi with cellulases or hemicellulases for their secretion have been described [7–9]. The display of cellulases on bacterial cell walls has also been reported in *Escherichia coli* and *Bacillus subtilis* [10–12]. In order to develop a consolidated bioprocessing organism, cellulases or xylanases have also been displayed on yeast cells [13–17].

Formation of anchored cellulosome complexes on the cell wall has been studied as well in *Saccharomyces cerevisiae* [18–20]. Assembly of mini-cellulosomes was achieved either by incubating the yeast with purified dockerin-containing cellulases [18, 19] or by direct *in vivo* co-expression of the enzymes [20]. Subsequently, several authors succeeded in assembling cellulosome complexes either by incubating *E. coli* cells lysates containing the enzymes [21, 22], or by cultivating a consortium of cells secreting the enzymes [23]. Cellulosome-inspired complexes were also grafted onto the cell surface of *Lactococcus lactis* [24]. Recently, another publication demonstrated that it is possible to use sortase enzymes to attach a mini-cellulosome to the surface of *B. subtilis* [25]. Secretion and assembly of cellulosomes in *Clostridium acetobutylicum* were also achieved [26].

The lactobacilli are a group of bacteria with extensive industrial applications which include production of commodity chemicals, flavor compounds and vitamins [27] *.* Among the lactobacilli, *L. plantarum* possesses many singular advantages towards biomass deconstruction. In nature, this bacterium is prominent in plant biomass environments, and is frequently used in the food industry and agricultural applications. Its high acid tolerance renders it less sensitive to contamination, and offers a valuable advantage in biomass degradation, as some plant biomass pretreatments generate acidic conditions. Furthermore, *L. plantarum* is capable of utilizing both hexose and pentose sugars, thus potentially providing a natural platform for exploiting more of the biomass degradation products in favor of downstream commodity production. The concept of engineering *L. plantarum* to produce ethanol from plant biomass is very tempting as this bacterium possesses a high tolerance to ethanol (up to 13% (v/v)), under conditions of low pH (in the range 3.2 to 4) [28]. Altogether, this bacterium could represent a competitive alternative to other types of microbial systems (*Clostridium thermocellum*, *S. cerevisiae* or *E. coli*), engineered for this purpose [19, 29]. Likewise, *L. plantarum* is an appealing potential producer of other important biochemicals and biofuels, such as butanol, lactic acid (the precursor for polylactic acid) and other chemicals [27], due to its inherent traits and its genetic and metabolic potential.

The *L. plantarum* genome contains 55 genes encoding for 18 glycoside hydrolases families [1] but none of them are strict cellulases or xylanases. We recently demonstrated that we could complement the set of enzymes of *L. plantarum* by introducing a potent cellulase and a potent xylanase, and obtain synergistic degradation of hypochlorite pretreated wheat straw by a consortium of two separate *L. plantarum* cells*,* engineered to secrete these enzymes [30]. In that study, we separated the expression of each enzyme by incorporating the genes for the cellulase and xylanase into different *L. plantarum* cells. By doing so, we decreased the burden of the cellular machinery of each strain, thereby maximizing its ability to grow, express and secrete each enzyme. Such spatial differentiation is a common strategy in nature when performing a certain metabolic process, thus allowing higher efficiency in a cell consortium as each cell is likely to have greater specialization [31]. Apart from using cell consortia to perform a complex metabolic task such as lignocellulose degradation, spatial differentiation could be harnessed to assemble large enzymatic complexes on cell walls by expressing each component from different cells. Such ‘intercellular complementation’ was originally employed for the formation of single-enzyme mini-cellulosomes in a *B. subtilis* host cell system [32].

Using this approach, in the present article we have constructed functional designer cellulosomes on the cell surface of *L. plantarum* using ‘designer cellulosome’ technology to mimic the architecture of the cellulosome complexes and specifically control their enzyme composition [33–39]. In addition, we displayed the lignocellulolytic enzymes on the *L. plantarum* cell wall.

The cell-consortium approach was thus employed to assess the significance of enzyme localization in biomass deconstruction by comparing the three different enzymatic paradigms: (i) a secreted enzyme system, (ii) enzymes anchored to the bacterial cell surface and (iii) enzymes integrated into cellulosome complexes.

## Results

**Figure 1:**
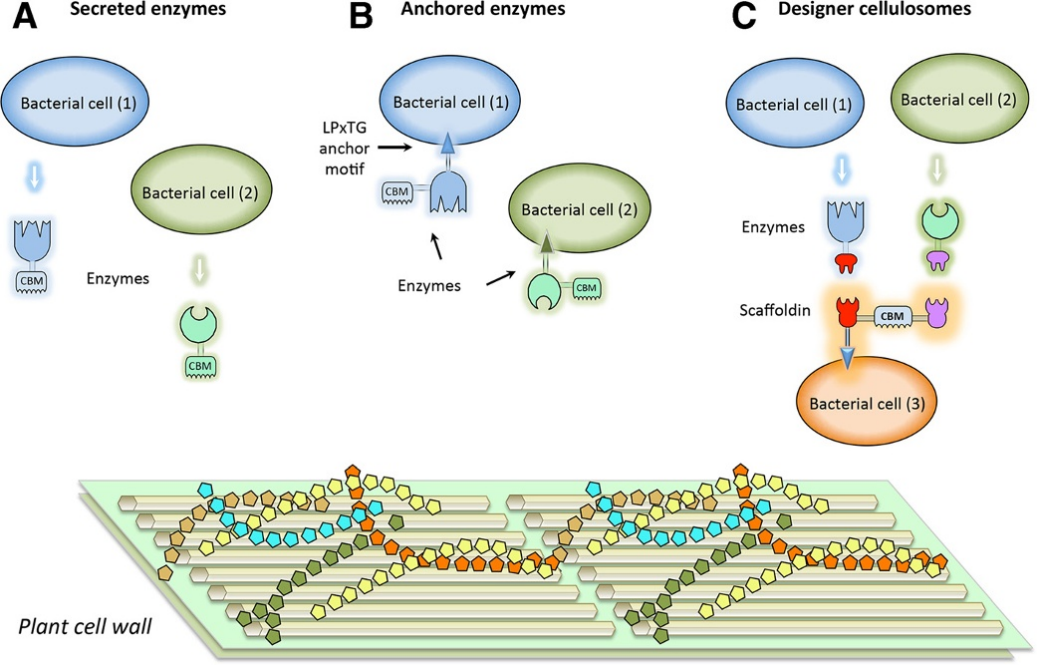
**Schematic representation of the three enzymatic paradigms examined in this study. A**. Coculture of two *L. plantarum* strains secreting the Cel6A cellulase and the Xyn11A xylanase. **B**. Coculture of two respective *L. plantarum* strains producing the Cel6A cellulase and the Xyn11A xylanase anchored to the bacterial cell wall. **C**. Coculture of two respective *L. plantarum* strains secreting the dockerin-containing 6A-*t* cellulase and 11A-*a* xylanase and an additional strain producing the Scaf · AT scaffoldin on the bacterial cell wall, resulting in the formation of the designer cellulosomes on the latter cell. The enzymes (arrows) and scaffoldin components are color-coded according to that of the parent cell. CBM – carbohydrate binding module.

The principles for the design of the three enzymatic paradigms examined in this study are presented in Figure 1. In the secreted enzyme paradigm (Figure 1A), a cell consortium of two strains, each producing either a cellulase or a xylanase, was established. In the anchored enzyme paradigm (Figure 1B), the two strains anchor the same two enzymes individually on their respective cell surfaces. In the designer cellulosome paradigm (Figure 1C), the cell that anchors the scaffoldin on the cell wall integrates into the complex the two distinct enzymes produced by ‘helper’ strains.

### Enzyme secretion by *L. plantarum*

For the first paradigm we used secretion of the cellulolytic enzymes by *L. plantarum*, which was the subject of a previous study [30]. Cellulase concentrations at OD_600_ = 1 were estimated at 0.33 nM and 0.27 nM for the Lp1 (Leader peptide) and Lp2 secretion plasmids, respectively. For the xylanase these values were estimated at 2.7 nM and 3.3 nM, respectively (Table 1). As reported, the concentrations of the secreted enzymes in the different cultures were calculated by comparing the extracellular fraction to serial dilutions of purified enzymes, both by dot-blot analysis and by measuring reducing sugar formation on phosphoric acid-swollen cellulose (PASC) or xylan substrates. The concentrations, calculated by both methods, were similar for both enzymes, suggesting that the major portion of the secreted enzymes is functional and that the expression and secretion processes do not substantially affect their activity. The same method was used for calculating the concentrations of the other enzymes produced in the present study (anchored enzymes and secreted dockerin-containing enzymes) and for ensuring their full integrity (Additional file 1: Figure S1, Additional file 2: Figure S2, Additional file 3: Figure S3 and Additional file 4: Figure S4).

**Table 1:** **Concentrations (in nM) of the enzymes and scaffoldin produced by transformed** ***L. plantarum***

	Lp1	Lp2	cwa1	cwa2	cwa3
Cellulase (+dockerin)	*0.33 (0.25)*	0.27 (0.22)	*0.133*	0.133	nd
Xylanase (+dockerin)	2.7 *(2.5)*	*3.3* (1.7)	*0.08*	0.06	0.03
Scaffoldin	-	-	1	*1.63*	-

nd: not detected.

Values in italics correspond to the plasmids chosen for activity experiments.

### Enzyme anchoring by *L. plantarum*

The second paradigm consisted of anchoring the cellulolytic enzymes to the cell wall of *L. plantarum*. The presence of anchored enzymes on the cells was observed by dot-blot assay using specific antibodies against each enzyme (Additional file 3: Figure S3 and Additional file 4: Figure S4). The enzymes were visible on the cells carrying the cell wall anchoring (cwa) plasmids cwa1, cwa2 and cwa3. No enzymes were detected on the cells of strains carrying the expression plasmid lacking the anchoring signal (Additional file 3: Figure S3 and Additional file 4: Figure S4). The cellulase concentrations at OD_600_ = 1 were estimated at 0.133 nM for the cwa1 and cwa2 anchoring plasmids (Additional file 3: Figure S3), and could not be detected for the strain carrying the cwa3 plasmid (Table 1). For the xylanase, these values were estimated at 0.08, 0.06 and 0.03 nM for the cwa1-, cwa2- and cwa3-carrying strains, respectively (Additional file 4: Figure S4 and Table 1).

### Designer cellulosome assembly on *L. plantarum*cells

In order to assemble designer cellulosomes on the cell surface of *L. plantarum* we first focused our endeavors on the construction and anchoring of the scaffoldin, the architectural module of the cellulosomes, and subsequently on the design and secretion of two matching cellulosomal enzymes.

#### Active scaffoldin expression by *L. plantarum*

In order to assemble the simplest type of designer cellulosome involving only two enzymes (in this case a cellulase and a xylanase), chimeric Scaf · AT was designed to bear two different cohesins and a cellulose-binding carbohydrate binding module (CBM), where A represents an *Acetivibrio cellulolyticus* cohesin, and T, a *C. thermocellum* cohesin. The latter cohesins interact specifically with their dockerin-bearing enzyme counterparts, which ensures that each scaffoldin will bind one molecule of each enzyme. This arrangement enables the precise control of the stoichiometry and enzyme organization on the scaffoldin. This particular scaffoldin was initially produced in *E. coli* and its cohesin specificities for the chimeric dockerin-bearing enzymes were examined semi-quantitatively by a sensitive enzyme-linked affinity assay in microtiter plates [40]. Both cohesins specifically bound their respective dockerins and did not bind other, non-matching dockerin-bearing molecules (data not shown).

**Figure 2:**
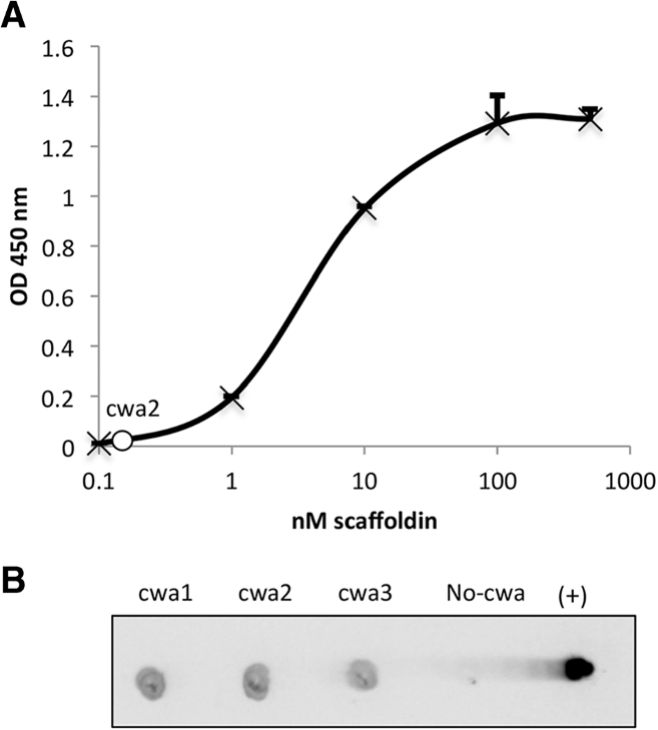
**Expression and functionality of the anchored scaffoldin. A**. Determination of scaffoldin concentration by whole-cell ELISA. Plates were coated with a suspension of whole bacterial cells carrying Scaf · AT in cwa2 (OD_600_ = 0.1) or increasing concentrations of the pure recombinant Scaf · AT (0.01 to 500 nM). The graph shows the OD_450_ of each concentration for the calibration curve in black, and the white circle in the insert represents the concentration of Scaf · AT in the cwa2 carrying strain. Cultures were performed in triplicate, and standard deviations are indicated. **B**. Cellulose binding ability of the scaffoldin. Cellulose-chip binding by strains carrying cwa1, cwa2, cwa3 or No-cwa grown on MRS without proteose peptone. The pure recombinant Scaf · AT scaffoldin served as a positive control. cwa – cell wall anchor.

After verifying the specificity characteristics, the scaffoldin was then expressed and anchored to the cell wall of *L. plantarum* using three different lengths of anchor signals, cwa1, cwa2 and cwa3 [41]. The presence of the scaffoldin on the cell surface was assessed by dot-blot assay using a specific antibody against the CBM (Additional file 5: Figure S5). The number of scaffoldins on the cells was calculated using whole-cell ELISA, by comparing the intensity to known concentrations of the pure recombinant Scaf · AT (Figure 2A). The molarities of the scaffoldin on the cells in cwa1 and cwa2 were determined at 1 and 1.63 nM, respectively (Table 1).

**Figure 3:**
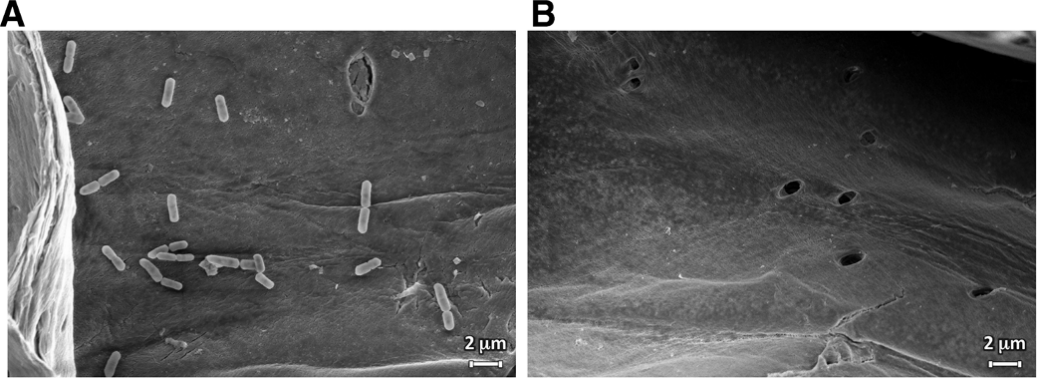
**Scanning electron microscopy of induced culture of** ***L. plantarum*** **harboring scaffoldin Scaf · AT in cwa2-carrying strain on the cell wall (A) or wild-type strain (B) attached to hypochlorite pretreated wheat straw fibers.** Bar, 2 μm.

When grown on Lactobacillus-MRS bacterial growth medium, the cells harboring the scaffoldin failed to bind to cellulose on a regenerated cellulose-coated slide, suggesting the presence of a binding-inhibitory component within the MRS. Therefore, the effect of each component of the MRS medium was evaluated for its possible inhibitory effect on the pure recombinant Scaf · AT to bind to cellulose (Additional file 6: Figure S6). It was observed that both the presence of polysorbate 80 (Tween 80) and proteose peptone diminished the ability of the CBM to bind to cellulose. Thus, we omitted them from the medium and measured the bacterial growth and attachment to cellulose in their absence. The absence of proteose peptone from the medium had no effect on bacterial growth, whereas the generation time was reduced without Tween 80 (Additional file 7: Figure S7). Furthermore, the cells expressing the scaffoldin recovered the ability to bind to cellulose, when grown in the medium without proteose peptone (Figure 2B), which was selected for further experiments. The scaffoldin-expressing strains with the three different cell wall anchors were examined for their ability to attach to cellulose. The strain harboring the cwa2 plasmid exhibited higher affinity to cellulose than the strains harboring cwa1 and cwa3 (Figure 2B) and therefore was selected for further experiments. We further validated the attachment via the scaffoldin of these induced bacterial cells to hypochlorite pretreated wheat straw fibers by scanning electron microscopy. This analysis revealed that the cwa2 scaffoldin-expressing cells attached readily to the plant fiber as opposed to the wild-type strain which failed to attach (Figure 3).

**Figure 4:**
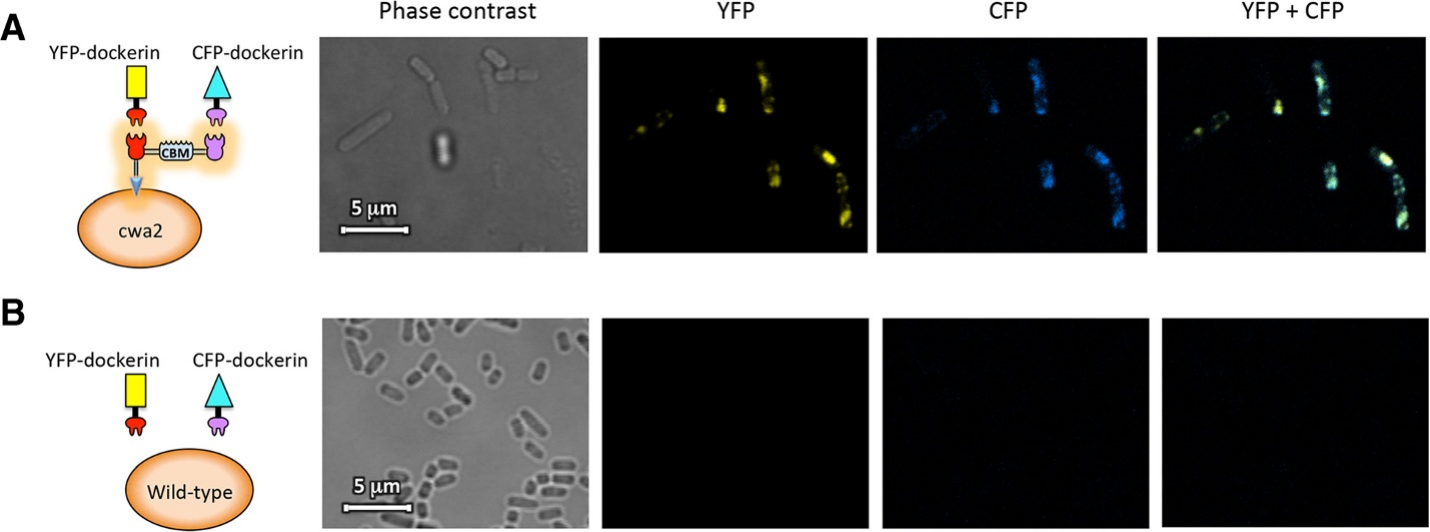
**Confocal fluorescence microscopy images of Scaf · AT in cwa2-carrying strain (A) or wild-type cells (B) after binding interaction with mVenus yellow fluorescent protein (YFP) and mCerulean cyan fluorescent protein (CFP) fluorophores fused to respective dockerin modules.** Bar, 5 μm.

The functionality of the displayed scaffoldin was then assayed. We validated the ability of the scaffoldin to co-localize two different dockerin-containing proteins by using a reporter system composed of fluorescent proteins fused to dockerin modules. Localization and activity of the cohesin sites on the scaffoldin were assessed by fluorescent labeling of the transformed *L. plantarum* cells, using two different dockerin-containing fluorophores - mVenus yellow fluorescent protein (YFP) containing an *A. cellulolyticus* dockerin and mCerulean cyan fluorescent protein (CFP) containing a *C. thermocellum* dockerin. As shown in Figure 4, while no fluorescence was observed on the wild-type bacteria, in the transformed *L. plantarum* cells, the two reporter proteins were co-localized on the bacterial cells, thus demonstrating that the cohesins on the scaffoldin displayed on the bacterial cell wall are both active and that the attachment of two different cellulolytic enzymes is feasible.

#### Design and expression of cellulosomal enzymes by *L. plantarum*

After validating the functionality and attachment of the scaffoldin on the cell wall, we continued to construct the matching cellulosomal enzymes. We used two potent cellulolytic enzymes selected from *Thermobifida fusca*: cellulase Cel6A and xylanase Xyn11A, previously used in designer cellulosomes [36, 38, 42] and successfully secreted in *L. plantarum* [30]. Each enzyme was fused to a dockerin of divergent specificity to match the chimeric scaffoldin. The chimeric cellulase, 6A-*t* was designed to contain a dockerin derived from the *C. thermocellum* xylanase Xyn10Z at the C-terminus of the catalytic module, replacing the original cellulose-binding CBM [43]. In chimaera 11A-*a*, an alternative dockerin from *A. cellulolyticus* was appended at the C-terminus of the original Xyn11A, whereby the original catalytic module and the essential cellulose and xylan-binding CBM were both retained [36].

In order to ease the burden on a single expressing strain, we used a biomimicry approach in which the dockerin-containing enzymes are expressed and secreted from helper strains to act as a cell consortium. These enzymes were expressed in *L. plantarum* strains using Lp1 and Lp2 secretion plasmids [30, 44]. The presence of the secreted enzymes in culture supernatant was observed by Western blotting using specific antibodies against each enzyme (Additional file 8: Figure S8). The enzymes were visible in the extracellular fraction of the strains carrying the Lp1 and Lp2 secretion plasmids, and the observed bands corresponded well to their theoretical masses. Culture supernatants of secreting strains were able to degrade PASC (endoglucanase 6A-*t*) or xylan (xylanase 11A-*a*) substrates. These activities were used to determine the molarities of the enzymes in the cultures by comparing them to those of pure recombinant enzymes (Additional file 1: Figure S1 and Additional file 2: Figure S2). The molarities were determined at 0.25 and 0.22 nM for endoglucanase 6A-*t* in Lp1 and Lp2, respectively; and 2.5 and 1.7 nM for xylanase 11A-*a* in Lp1 and Lp2, respectively (Table 1). No extracellular enzymes were detected by Western blotting in the supernatant fluids of strains carrying the expression plasmid lacking the secretion peptide. Additionally, supernatant fractions of cultures expressing the respective enzymes intracellularly exhibited negligible hydrolytic activity on those substrates, validating that the activity of the secreting strains is due to extracellular enzymes only.

#### Functionality of the designer cellulosome paradigm

**Figure 5:**
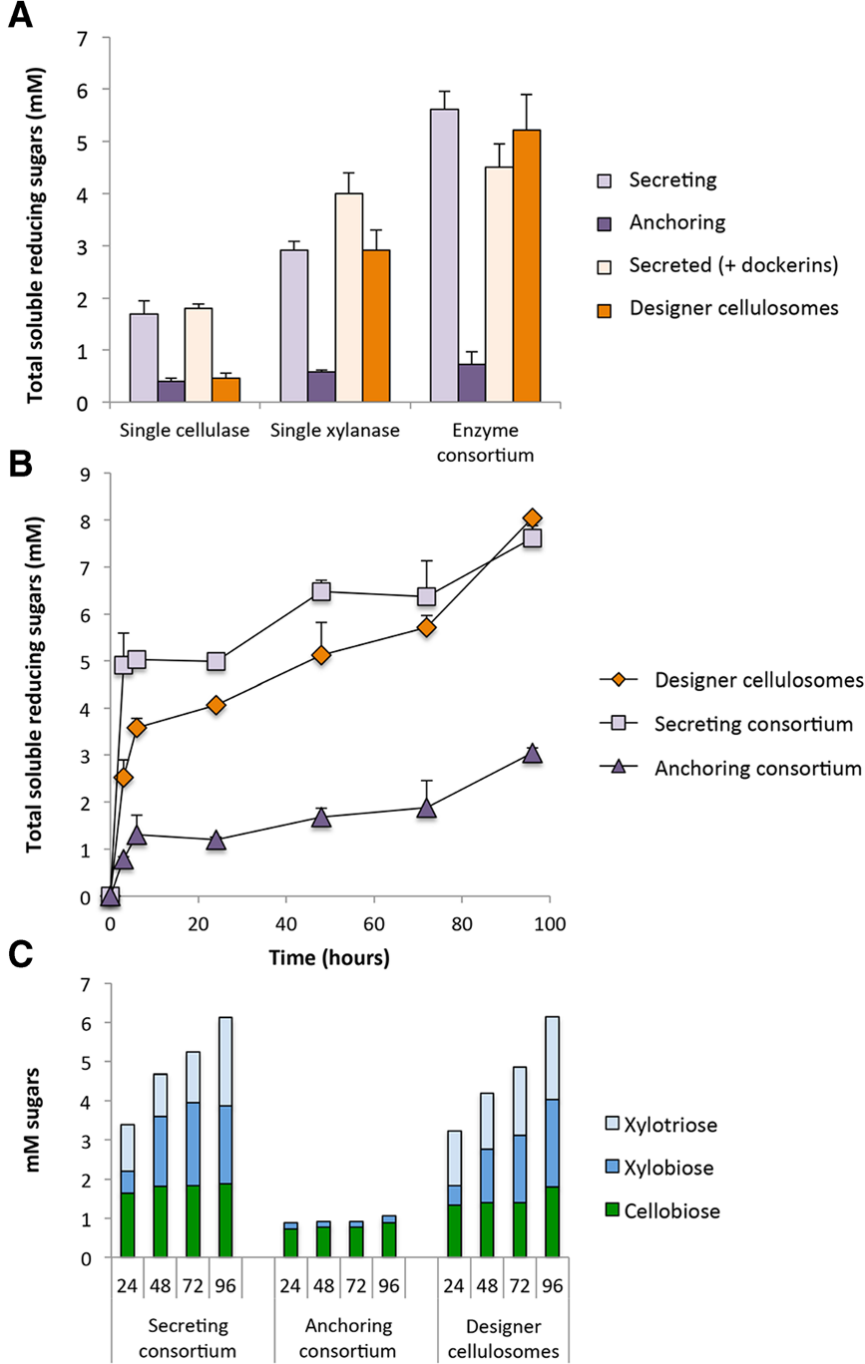
**Enzyme activity studies. A**. Comparative enzymatic activity of secreted or anchored cellulases and xylanases either individually or in a cell consortium. The concentration of hypochlorite pretreated wheat straw (dry matter) in the reactions was 40 g/l, and enzymes (either secreted or anchored) were added at 5 nM. Enzymatic activity is defined as mM soluble reducing sugars following a 24-hour reaction period at 37°C and pH 5. Each reaction was performed in triplicate and standard deviations are indicated. **B**. Kinetics studies of hypochlorite pretreated wheat straw hydrolysis by the secreted enzymatic consortium (squares) versus the designer cellulosome (diamonds) or the anchored enzymatic consortium (triangles). The concentration of hypochlorite pretreated wheat straw (dry matter) in the reactions was 40 g/l, and enzymatic consortium was added at 5 nM. Enzymatic activity is defined as mM soluble reducing sugars following a 3, 6, 24, 48, 72 or 96-hour reaction period at 37°C and pH 5. Each reaction was performed in triplicate and standard deviations are indicated. **C**. Soluble sugar production in mM as determined by HPLC analysis following digestion of hypochlorite pretreated wheat straw over a 24, 48, 72 or 96-hour incubation period by the designated cell consortium. Glucose, xylose, arabinose and cellotriose were not detected.

After validating the activity of each of the cellulosomal components separately, we examined the functionality of the overall complex anchored to the bacterial cell wall. Following our cell-consortium design, we used a secreting strain for each of the enzymes, each of which was designed to dock onto the cell wall-anchored scaffoldin using divergent cohesin-dockerin interactions (Figure 1). The activity of the fully assembled designer cellulosomes was measured on a hypochlorite pretreated wheat-straw substrate after eliminating the excess secreted enzymes in the supernatant fraction. This assay provided evidence for the functionality of the cell wall-anchored designer cellulosomes (Figure 5A, orange bars).

As a negative control for designer cellulosome formation, the strains transformed with the control plasmids (No-Lp) containing the enzymes, were also co-cultured either with the strain containing the anchored scaffoldin (cwa2) or the scaffoldin expressed internally (No-cwa), and assayed for xylan, cellulose and hypochlorite pretreated wheat straw degradation. These controls exhibited negligible enzymatic activity (Additional file 9: Figure S9), indicating that the activities detected above reflect properly secreted enzymes and do not originate from cell lysis, and that the designer cellulosomes were assembled on the cell surface.

### Activity of the enzymatic paradigms

#### Comparison of hypochlorite pretreated wheat straw degradation by single enzymes expressed by L. plantarum

We first measured the activity of individual enzymes belonging to the three different paradigms, secreted (with or without dockerin), anchored directly to the cell wall or attached to the cell wall-anchored scaffoldin. Secreted single cellulases with or without dockerin modules had enhanced enzymatic activity on hypochlorite pretreated wheat straw compared to their anchored counterparts (Figure 5A). The secreted xylanase containing the dockerin module was the most efficient xylanase in hypochlorite pretreated wheat straw degradation, whereas the secreted xylanase or the enzyme bound to the scaffoldin were slightly less efficient, and the anchored enzyme was about 7-fold less efficient (Figure 5A).

After measuring the activity of the different enzymatic strategies we assayed their ability to retain activity over time. The xylanases retained their activity at 37°C in all the examined expression strategies whereas differences were noted among the cellulases (Table 2). The cellulases anchored either directly to cell wall or via the scaffoldin maintained higher activity levels after 24 hours of incubation at 37°C than the secreted ones (Table 2).

**Table 2:** **Stability in activity (in% ± standard deviation) of single cellulase and xylanase at 37°C for a 24-hour period**

Cel6A		6A-*t*		Xyn11A		11A-*a*	
(wild-type, CBM-containing)		(chimeric, dockerin-containing)		(wild-type, CBM-containing)		(chimeric, dockerin-containing)	
Secreted	Anchored	Secreted	Designer cellulosome	Secreted	Anchored	Secreted	Designer cellulosome
58 ± 2	88 ± 8	6 ± 3	98 ± 3	100 ± 2	95 ± 6	100 ± 2	100 ± 0

#### Hypochlorite pretreated wheat straw degradation by dual enzyme systems

The secreted enzyme consortium exhibited the highest enzymatic activity of hypochlorite pretreated wheat straw degradation, while the anchored enzyme consortium had the lowest enzymatic activity; about 8-fold less than the secreted enzyme consortium. The designer cellulosomes containing the scaffoldin had slightly reduced activity compared to the secreting consortium (Figure 5A). The secreted dockerin-containing enzymes (without the strain carrying the scaffoldin) were slightly less active compared to the designer cellulosomes, indicating the advantage of placing the two enzymes in close proximity via the chimeric scaffoldin.

The complexity of the substrate may vary the enzymatic rates of degradation over time. Thus, we measured the kinetics of substrate degradation by the three different paradigms (Figure 5B). Our examination exhibited the typical biphasic slopes resulting from the degradation of swollen and crystalline parts of the insoluble substrate [45, 46] for the three enzymatic paradigms. The designer cellulosome and the secreting consortia were far more efficient than the anchoring consortium throughout the time range of the experiment. In the short term (3 hours), the secreting consortium was about 2-fold more efficient than the designer cellulosomes. Afterwards, the sugar production rate of the designer cellulosomes increased significantly, and the difference between the designer cellulosomes and the secreting consortium decreased as incubation time was prolonged, until similar levels of released soluble sugars were finally reached (96 hours). This phenomenon can be linked to the difference in retention of enzymatic activity, whereby the enzymes bound to the scaffoldin appear more stable than those secreted in the medium.

The analysis of the degradation products revealed that designer cellulosomes and the secreting consortium produced xylotriose, xylobiose and cellobiose (Figure 5C) whereas only cellobiose and very low levels of xylobiose were detected in the anchored consortium. The amount of released cellobiose remained stable or slightly increased over time, while the amount of xylotriose and xylobiose, products of xylanase activity, increased gradually over time for the secreting and designer cellulosome consortia.

## Discussion

*L. plantarum* can be considered a prospective organism of choice for bioprocessing of plant biomass, as it can lead to the production of valuable products such as bioethanol or polylactic acid [47, 48]. Thus, we previously complemented it with the capacity for fibrolytic activity via secretion of a cellulase and xylanase, using a cell-consortium approach. Here we extended this approach by anchoring the two enzymes to the cell walls of *L. plantarum* strains and in parallel provided an alternative strategy by constructing a simple but functional cellulosome complex assembled *in vivo* on the cell surface of *L. plantarum,* significant steps towards the realization of consolidated bioprocessing (CBP) of plant biomass [49]. The choice of this particular bacterial platform to serve as a biorefinery industrial organism was also motivated by the fact that providing it with cellulolytic activities would not involve interference with the cell metabolism.

In order to construct the cellulosomal complexes we adopted a cell-consortium approach whereby each *L. plantarum* strain expresses an individual cellulosomal component, similar to the approach pursued for the consortia of single enzyme expressing cells (secreted or anchored). Using this simple approach, each *L. plantarum* strain has an identical genome, except for the expressed enzyme (or scaffoldin). Therefore an essentially homogeneous culture of the same bacteria with a variety of functions is obtained, while each individual strain has a decreased load on its cellular machineries. In nature this type of spatial differentiation strategy is very common in the prokaryotic world where different cells collaborate to perform a unified task from which they all benefit [31]. In the rumen microbial ecosystem an assortment of microbial species expressing different enzymatic functions act in harmony for the direct degradation of the complex fiber substrate. Moreover, the use of hydrogen by methanogenic archaea reduces the concentration of that inhibitory product and contributes to enhance fiber degradation by hydrogen-producing cellulolytic bacteria [50]. By mimicking nature, we compared the three highly prevalent cellulolytic strategies of enzyme localization.

In the cellulosome paradigm, we were successful in providing *L. plantarum* with cellulose attachment abilities via the CBM module on its cell wall-anchored scaffoldin. Nevertheless, a relatively low cellulose-binding ability (approximately 30%) of the bacterium transformed with the scaffoldin was observed, as compared to the reported *C. thermocellum* adherence to microcrystalline cellulose (approximately 70 to 80%) [51] and to *E. coli* expressing a CBM at its cell surface [52]. This may be attributed to a relatively low concentration of the scaffoldin on the bacterial cell wall. Furthermore, lower concentrations of both enzymes were measured on the bacterial cell wall in the anchored paradigm as compared to their secreted counterparts. In this context it was reported that in yeasts, challenges remain regarding the amount of protein that can be displayed on the yeast surface without destabilizing the cell [21, 22, 53]. Those authors developed different strategies using cellulosome-like architectures to augment the number of enzymes on the cell surface. Alternatively, accessibility of the scaffoldin-borne CBM may be limited. This possibility may be rectified by use of a protracted linker segment in the scaffoldin that would further distance the functional modules (CBM, cohesins) from the bacterial cell surface. Another alternative would be to employ a scaffoldin with the CBM at the N-terminus of the molecule, which could supply it with increased steric freedom for interaction with the substrate.

The secreting consortium and the designer-cellulosome consortium exhibited similar levels of substrate degradation activity for both enzymes (Figure 5C). Secreted enzymes had a slight advantage over the designer cellulosome complexes in degrading the hypochlorite pretreated wheat straw substrate. However, the individual secreted enzyme activities (Table 2) decreased with incubation time whereas the activities of the enzymes bound to the designer cellulosomes remained constant, suggesting an advantage of the latter over time. In addition, enzyme adsorption to lignin may be more crucial in free enzymes, as it was recently demonstrated that cell wall-anchored enzymes are less subjected to irreversible lignin adsorption than secreted enzymes [15]. Likewise, the replacement of the original CBM by a dockerin in the *T. fusca* cellulase could also reduce cellulase adsorption by lignin as it was demonstrated that removal of the CBM in a cellulase resulted in an enhancement of degradation of lignin-containing substrates, by avoiding the unproductive binding of the cellulase to lignin [54].

The single anchored enzymes exhibited significantly lower levels of enzymatic activity as compared to the other enzymatic paradigms, particularly the xylanase. This may be a function of allosteric hindrance due to the proximity of the enzymes to the bacterial cell. In designer cellulosomes the scaffoldin increases the distance from the cell, and while this restored the activity of the xylanase, the same is not true for the cellulase (Figure 5A). Attachment of the cellulase to the cell surface, either by anchoring it to the cell wall or by attaching it to the scaffoldin, diminished its activity. However, combining the cellulase and xylanase on the scaffoldin resulted in a synergistic effect, similar to what we previously observed for the secreting consortium [30]. This effect was not apparent in the anchoring consortium, suggesting a proximity effect that could account for the observed synergism. The major difference between designer cellulosomes and the anchoring cell consortium is that these two simple enzymes are either in close proximity on the same bacterial cell in the case of the former, or anchored to two different bacterial cell populations in the latter strategy. This distinction appears to be of great importance in terms of enzymatic activity. In this respect, enzymes on designer cellulosomes are similar to secreted enzymes, which could also be in close contact. This enables the cellulase accessibility to cellulose exposed by the action of the adjacent xylanase.

The designer cellulosome paradigm embodies advantages of both the secreted and the anchored paradigms. It allows for close proximity of different enzymes while keeping the enzymes, and therefore their products as well, within immediate range of the cell itself. Moreover it enables us to specifically control the stoichiometry of the enzymes and their organization on the cell surface. Employment of a same-cell consortium (a single bacterial species; in this work, *L. plantarum*) for production of different cellulosome elements reduces the burden of protein production, secretion and anchoring for the bacterium. At the same time it ensures that all community members will react to medium components similarly - in this case benefit from the products released into the medium by the enzymatic activity, and be more resistant to downstream bioprocessing products such as ethanol or polylactic acid.

## Conclusions

As opposed to other bacteria, in which designer cellulosomes or glycoside hydrolases have been displayed or secreted (such as *E. coli* or *B. subtilis*), *L. plantarum* is an acknowledged ‘generally regarded as safe’ (GRAS) bacterium with a large variety of both proven and potential industrial applications. Further improvement of *L. plantarum* as a lignocellulolytic bacterium could therefore lead to optimal biomass bioprocessing schemes, appropriate for immediate industrial acceptance.

In using a cell-consortium approach for this type of study we are essentially mimicking nature, since similar phenomena are occurring whereby different bacterial cells in a given ecosystem produce enzymes with distinct complementary specificities, which contribute to the overall welfare of the community. The biomimicry approach, based on specified cell consortia, allows for stoichiometric control and spatial optimization of the different expressed components. These cell consortia may thus be more amenable for consolidated bioprocessing strategies than a single organism, especially for cellulose breakdown with respect to potential production of subsequent chemical commodities [31]. Such a spatial differentiation approach via dedicated cell consortia may thus become the method of choice for deployment of metabolic engineering for complex bioprocessing efforts.

## Materials and methods

### Cloning

Wild-type enzymes Cel6A and Xyn11A were cloned from *T. fusca* genomic DNA as described previously [36, 55]. The enzyme constructs in pET28a were designed to contain a His-tag for subsequent purification.

For expression and secretion in *L. plantarum*, the glycoside hydrolases were cloned in the modular secretion plasmids pLp_2145sAmy and pLp_3050sAmy [44] (Lp1 and Lp2, respectively) by replacing the amylase gene in these plasmids by an appropriately amplified gene fragment, using either *Sal*I or *Xho*I (*Sal*I is compatible with *Xho*I) and *Hin*dIII restriction sites as described previously [30].

For anchoring to the cell wall of *L. plantarum*, the glycoside hydrolases were cloned into the modular *p*Lp_0373sOFA anchoring plasmids (cell wall anchored 1, 2 and 3: cwa1, cwa2 and cwa3, respectively) [41]. The Cel6A encoding gene was amplified using 5’-atatatCTCGAGgcatcccccagacctcttcgcg-3’ and 5’-atatatACGCGTctccaggctggcggcgcagg-3’ primers (*XhoI* and *MluI* sites in capital letters), and the Xyn11A gene were amplified using 5’-accgatGTCGACgccgtgacctccaacgagac-3’ and 5’-actgatGCGCGCgttggcgctgcaggacaccg-3’ primers (*SalI* and *BssHII* sites in capital letters). The genes were ligated into the anchoring plasmids *p*Lp_0373sOFAcwa1, *p*Lp_0373sOFAcwa2 and *p*Lp_0373sOFAcwa3.

Dockerin-containing chimaeras, 6A-*t* and 11A-*a*, and the recombinant scaffoldin Scaf · AT were cloned in pET28a (Novagen Inc., Madison, United States) with a His-tag as described previously [36, 43]. The chimeric cellulase, 6A-*t*, was amplified from *p*6A-*t* using 5’-tgaatCTCGAGgccaatgattctccgttctac-3’ and 5’-tatctAAGCTTttatccggggaactctgtaa-3’ primers (*Xho*I and *Hind*III sites in capital letters) and ligated into the Lp1 and Lp2 secretion plasmids. The chimeric xylanase 11A-*a* was cloned using 5’-tagtaGTCGACgccgtgacctccaacgagac-3’ and 5’-attacAAGCTTttattcttctttctcttcaac-3’ primers (*Sal*I and *Hind*III sites in capital letters), and ligated into the secretion plasmids Lp1 and Lp2.

The chimeric scaffoldin, Scaf · AT was cloned from the *p*Scaf · AT using the forward primer 5’- tagtaGTCGACgatttacaggttgacattgg-3’ and reverse primer 5’- attacACGCGTtatatctccaacatttactc-3’ (*Sal*I and *Mlu*I sites in capital letters, New England Biolabs Inc., Massachusetts, United States) and inserted (T4 DNA ligase: Fermentas UAB, Vilnius, Lithuania) in the *p*Lp_0373sOFA anchoring plasmids [41].

The scaffoldin, the wild-type enzymes and the chimeric enzymes were also cloned into pSIP407 plasmid (referred to as No-Lp or No-cwa) [56] for control of intracellular expression (non-secreted and not directed to the cell wall), using the above listed primers but with *Nco*I and *Xho*I as restrictions sites. In the case of 11A-*a, BspH*I was used instead of *Nco*I, which cleaves in this DNA (*Bsp*HI is compatible with *Nco*I). PCR reactions were performed using Phusion High Fidelity DNA polymerase F530-S (New England Biolabs, Inc), and DNA samples were purified using a HiYield™ Gel/PCR Fragments Extraction Kit (Real Biotech Corporation, RBC, Taipei, Taiwan).

mCerulean cyan fluorescent protein (CFP) was cloned using 5’-acggccCCATGGtgagcaagggcgaggagc-3’ and 5’-cggatcGGTACCaacttgtacagctcgtccatg-3’ primers (*Nco*I and *Kpn*I sites in capital letters) and ligated to the dockerin Z of *C. thermocellum* amplified using 5’-cattagGGTACCtgaaagcagttccacaggtc-3’ and 5’-aatcatCTCGAGtccggggaactctgtaataatg-3’ primers (*Kpn*I and *Xho*I sites in capital letters) and to the linearized form of pET28a plasmid to form pCFP-*t*.

mVenus yellow fluorescent protein (YFP) was amplified using 5’-attccgCCATGGGCcatcaccatcaccatcacgtgagcaagggcgaggag-3’ and 5’-atgcctGGTACCcttgtacagctcgtccat-3’ (*Nco*I and *Kpn*I sites in capital letters) primer pair and ligated to the ScaA dockerin from *A. cellulolyticus* cloned using 5’-ttacaaGGTACCaaatttatatatggtgatgt-3’ and 5’-aatgtGAGCTCttattcttctttctcttcaa-3’ (*Kpn*I and *Sac*I sites in capital letters) primers and to the linearized form of pET28a plasmid to form pYFP-*a*.

Competent *E. coli* XL1 competent cells were used for transformation with the pET28a plasmids whereas *L. plantarum* plasmids were transformed in *E. coli* TG1 competent cells (Lucigen Corporation, Wisconsin, United States). Antibiotics used for positive clone selection and added in the medium were used at concentrations of 50 mg/ml kanamycin sulfate (Sigma Chem. Co, Missouri, United States) and 200 μg/ml erythromycin (Sigma Chem. Co, Missouri, United States) for *E. coli* and *L. plantarum* plasmids, respectively.

### Recombinant protein expression and purification

Wild-type enzymes (Cel6A and Xyn11A), dockerin-containing chimaeras, (6A-*t* and 11A-*a*) and the recombinant scaffoldins Scaf · AT were prepared as described previously [36, 43]. Briefly, the plasmids pCel6A, pXyn11A, p6A-*t*, p11A-*a* and pScaf · AT were expressed in *E. coli* BL21 (lDE3) pLysS cells and the His-tagged proteins were purified on a Ni-NTA column (Qiagen, Hilden, Germany), as reported earlier [42]. The purity of the recombinant proteins was tested by SDS-PAGE on 10% acrylamide gels and the fractions containing the pure recombinant protein were pooled and concentrated using Amicon centrifugal filters (Millipore, Molsheim, France). Protein concentration was estimated by absorbance (280 nm) based on the known amino acid composition of the protein using the Protparam tool [57]. Proteins were stored in 50% (v/v) glycerol at −20°C.

### Protein expression in *L. plantarum*

The electroporation of electro-competent *L. plantarum* WCFS1 was conducted according to the protocol by Aukrust and Blom [58]. Freshly inoculated cultures of *L. plantarum* WCFS1 harboring a pSIP-derived expression plasmid were induced at OD_600_ = 0.3 by adding the inducing peptide for sakacin P production (Caslo Laboratory, Lyngby, Denmark) [59] to a final concentration of 25 ng/ml and grown for 3 hours at 37°C (in MRS at 37°C, 10 μg/ml erythromycin). For co-culture experiments, strains producing either the cellulase or the xylanase, respectively, were mixed at stoichiometric ratios and then grown and induced in the same manner. For the designer cellulosome strategy, the MRS broth without proteose peptone was used and supplemented with 40 mM CaCl_2_. A consortium of bacteria producing the different proteins (the cellulase, the xylanase and the scaffoldin) were mixed at stoichiometric ratios and induced as described above.

### Western blotting

Proteins from the culture supernatants were separated on PAGE-SDS gel 10% acrylamide and transferred to a nitrocellulose membrane using Trans-Blot® Cell Mini (Bio-Rad Laboratories Ltd, California, United States). The membrane was then treated as described earlier [30].

### Affinity-based ELISA

The matching fusion-protein procedure of Barak *et al*. [40] was followed to determine cohesin-dockerin specificity of interaction for recombinant 11A-*a* and 6A-*t* and Scaf · AT.

### Whole-cell ELISA

MaxiSorp ELISA plates (Nunc A/S, Roskilde, Denmark) were coated with a suspension of whole bacterial cells (OD_600_ = 0.1 or increasing concentrations of the recombinant Scaf · AT (0.01 to 500 nM), in 0.1 M sodium carbonate coating buffer, pH9, for 1 hour at 37°C. The following steps were performed at 37°C with all reagents at a volume of 100 μl/well. The coating solution was discarded and blocking buffer (TBS (Tris buffer saline composed of Tris 3 g/l, NaCl 8 g/l, KCl 0.2 g/l adjusted to pH 7.4), 10 mM CaCl_2_, 0.05% Tween 20, 2% BSA) was added (1 hour incubation). The blocking buffer was discarded, and the primary antibody preparation (rabbit anti-CBM, diluted 1:3000 in blocking buffer) was added. After a 1-hour incubation period, the plates were washed three times with washing buffer (TBS, 10 mM CaCl_2_, 0.05% Tween 20), and the secondary antibody preparation (horseradish peroxidase (HRP) -labeled anti-rabbit antibody diluted 1:10,000 in blocking buffer) was added and incubated for 1 hour. The plates were again washed (four times) with washing buffer and 100 μl/well 3,3’,5,5’-Tetramethylbenzidine (TMB) + Substrate-Chromogen (Dako, Glostrup Denmark) was added. Color formation was terminated upon addition of 50 μl 1 M H_2_SO_4_ in each well, and the absorbance was measured at 450 nm using a microplate reader.

### Cellulose adherence assay

Induced cultures of Scaf · AT (in cwa1, cwa2, cwa3 or No-cwa) (1 ml of OD_600_ = 1) were washed and re-suspended in 100 μl of TBS. Volumes of 2 μl of each culture and the recombinant Scaf · AT were applied to a regenerated cellulose-coated slide (Advanced Microdevices Pvt, Ambala Cantt, India). To examine the effect of MRS components on the ability of the recombinant Scaf · AT scaffoldin to bind to the cellulose-coated slide, 0.5 mg/ml of the scaffoldin was subjected to 1-hour interaction at room temperature with each component concentrated as in Lactobacillus-MRS bacterial growth medium in a volume of 1 ml. Similarly, 2 μl of each solution was applied to the cellulose-coated slide. The slides were then treated as described above in the dot-blot assay section.

### Scanning electronic microscopy

After binding interaction with induced cultures of Scaf · AT (cwa2) or the wild-type strain, the hypochlorite pretreated wheat straw was washed rapidly in CaCo 0.1 M buffer, and the bacterial cells were then fixed with 3% paraformaldehyde and 2% glutaraldehyde in CaCo 0.1 M overnight at 4°C. The cells were washed with CaCo 0.1 M buffer and then several times with double-distilled water (ddw) and dehydrated in increasing concentrations of ethanol (30, 50, 70, 96 and 100%). The samples were then critical point dried in BAL-TEC CPD 030 and sputtered in gold palladium sputter coater (Edwards, Bolton, United Kingdom). The samples were then visualized using a secondary electron (SE) detector in a high-resolution Ultra 55 SEM (Carl Zeiss Microscopy GmbH, Oberkochen, Germany).

### Confocal fluorescence microscopy

A volume of 10 ml induced cultures (OD_600_ = 1) was centrifuged for 2 minutes at 4,500 g. The cells were washed quickly in TBS and fixed with paraformaldehyde (Sigma-Aldrich, Missouri, United States) diluted to 4% in TBS for 20 minutes. The cells were then washed and centrifuged for 2 minutes at 4,500 g, three times with TBS and once in TBS containing 1% Triton X-100 (Sigma-Aldrich, Missouri, United States). The cells were then re-suspended in 5% BSA diluted in TBS for blocking and dockerin-bearing fluorophores (*t*-CFP and YFP-*a*) were added in large excess. After a 1.5-hour interaction at room temperature, the cells were washed three times with TBS and mounted on slides with SlowFade® Gold anti-fade reagent (Life technologies, New York, United States) and 1.5% agarose. All microscopic observations and image acquisitions were performed with an Olympus IX-81 laser scanning confocal microscope (FV 500, Olympus Optical Co., Tokyo, Japan) equipped with an argon-ion laser (Showa Optronics Co. Ltd., Tokyo, Japan) and UPlanSApo 60 × 1.35 N.A oil objective (Olympus, Tokyo, Japan) . To observe CFP we used the 458 nm line for excitation and the BA480-495 emission filter. To observe YFP, we used 515 nm for excitation and the BA535-565 emission filter. For CFP and YFP detection, we used dichroic mirror (DM) 458/515. In all cases, where more than one color was monitored, sequential acquisition was performed. Transmitted light images were obtained using Nomarski differential interference contrast (DIC) microscopy (Olympus, Tokyo, Japan). The magnification can be increased by zooming the scanning laser beam on a smaller area of the object.

### Dot-blot assay

Induced cultures of 6A-*t*, 11A-*a* (both in Lp1, Lp2 or No-Lp) and Cel6A, Xyn11A and Scaf · AT (in cwa1, cwa2, cwa3 or No-cwa) were blotted (5 μl) on a Whatman Protean nitrocellulose membrane for blotting (Sigma-Aldrich, Missouri, United States). Non-specific protein interactions were blocked with 5% BSA (prepared in TBS-Tween 20, TBS-T) for 1 hour. The membrane was then rinsed twice with TBS-T for 1 minute. Rabbit antibody against either 6A-*t*, 11A-*a* or the CBM of Scaf · AT at dilutions of 1:2500 or 1:3000, were incubated with the membrane for 1 hour in TBS-T 1% BSA. The membrane was then rinsed twice with TBS-T for 1 minute. Secondary HRP-conjugated mouse anti-rabbit antibody, at a dilution of 1:10000 was subjected to a 1-hour interaction. The membrane was rinsed as described above and then rinsed twice with TBS + 1% Triton X-100 for 30 minutes. Blots were developed by incubating the membrane for 1 minute with equal amounts of solutions A and B of Enhanced chemiluminescence (ECL) (Ornat, Rehovot, Israel). Chemiluminescence was quantified using a luminescent image analyser, ImageQuant LAS 4000 Mini (Danyel Biotech, Rehovot, Israel).

### Activity assay

All assays were performed in triplicate. Enzymatic activity was determined quantitatively by measuring the soluble-reducing sugars released from the polysaccharide substrates by the dinitrosalicylic acid (DNS) method [60, 61]. DNS solution (150 μl) was added to 100 μl of sample, and after boiling the reaction mixture for 10 minutes, absorbance at 540 nm was measured. Sugar concentrations were determined using a glucose standard curve.

Prior to the enzymatic assays, culture supernatants (for secretion plasmids) were dialyzed in TBS containing 10 mM CaCl_2_ and cells (for anchoring plasmids) were washed with the same buffer by centrifugation re-suspension to eliminate the sugars present in the MRS medium (that would interact with the DNS reagent).

PASC (phosphoric acid swollen cellulose) degradation was assayed with recombinant Cel6A and 6A-*t* varying from 0 to 100 nM or 30 μl of concentrated supernatants of the cellulase expressing strains (Lp1, Lp2 or No-Lp) in a final volume of 200 μl (150 μl PASC (7.5 g/l), 50 mM acetate buffer pH 5.0). The suspensions were incubated at 37°C for 18 hours, and the reactions were terminated by immersing the sample tubes in ice water, before being centrifuged for 2 minutes at 14000 rpm to remove the substrate.

Xylanase assay mixture consisted of 100 μl buffer (50 mM citrate buffer pH 6.0, 12 mM CaCl_2_, 2 mM Ethylenediaminetetraacetic acid (EDTA) with recombinant Xyn11A and 11A-*a* (0 to 5 nM) or 30 μl of dialyzed supernatants of the xylanase expressing strains, Lp1, Lp2 or No-Lp. The reaction was commenced by adding 100 μl of 2% beechwood xylan (Sigma-Aldrich, Missouri, United States), suspended in 50 mM citrate buffer, pH 6.0, and the reaction was continued for 2 hours at 37°C. The reaction was stopped by transferring the tubes to an ice water bath and the tubes were centrifuged for 2 minutes at 14000 rpm.

Wheat straw (0.2 to 0.8 mm) (Valagro, Poitiers, France) was washed with deionized water as described previously [35, 62] and subjected to sodium hypochlorite treatment at a concentration of 12% at room temperature for 1 hour [63]. This pretreatment served to reduce the lignin content while preserving the cellulose and hemicellulose fractions in order to promote enzymatic degradation. The chemical composition of the pretreated wheat straw was 63% cellulose, 31% hemicellulose, and 3% lignin [63]. A typical assay mixture consisted of 1 ml reaction (50 mM acetate buffer pH 5.0, 12 mM CaCl_2_, 2 mM EDTA, 40 g/l hypochlorite pretreated wheat straw) containing 5 nM of concentrated supernatant fluids (using Amicon centrifugal filters (Millipore, Molsheim, France)) or washed cells (in TBS containing 1% Triton X-100), and reactions were incubated for 24 hours at 37°C. Similarly, kinetics studies were conducted and the reactions were incubated for 3, 6, 24, 48, 72 and 96 hours.

### Stability assay

The stability of each enzyme at 37°C was determined by incubating the enzyme (at 3 nM) without substrate over a 24-hours period, either at 4 or 37°C. The residual cellulase activity was calculated as the relative enzymatic activity of the enzyme incubated at 37°C compared to that of the enzyme incubated at 4°C, on PASC for a period of 18 hours at 37°C as described above. Similarly, the residual xylanase activity was calculated as the relative enzymatic activity of the enzyme incubated at 37°C compared to that of the enzyme incubated at 4°C, on beechwood xylan for a period of 2 hours at 37°C as described above.

### Sugar analysis

The sugars produced by designer cellulosomes, secreting or anchoring consortium were analyzed over time (24, 48, 72 and 96 hours) by HPLC (Agilent Technology 1260 Infinity with refractive index detector (Agilent Technology, California, United States)). The column (Aminex HPX-87H 300/7.8 mm (Bio-Rad, Hercules CA, United States) was eluted with 5 mM sulfuric acid, at 45°C and with a 0.6 ml/min flow. Calibration curves (0 to 30 mM of either glucose, cellobiose, cellotriose, xylose, xylobiose, xylotriose or arabinose) served to determine the amount of sugars.

## Electronic supplementary material

Additional file 1: Figure S1: Quantification of secreted cellulase 6A-*t* by dot-blot and enzymatic activity using known concentrations of the recombinant protein produced in *E. coli*. (TIFF 783 KB)

Additional file 2: Figure S2: Quantification of secreted xylanase 11A-*a* by dot-blot and enzymatic activity using known concentrations of the recombinant protein produced in *E. coli*. (TIFF 754 KB)

Additional file 3: Figure S3: Quantification of anchored cellulase Cel6A by dot-blot and enzymatic activity using known concentrations of the recombinant protein produced in *E. coli*. (TIFF 669 KB)

Additional file 4: Figure S4: Quantification of anchored xylanase Xyn11A by dot-blot and enzymatic activity using known concentrations of the recombinant protein produced in *E. coli*. (TIFF 704 KB)

Additional file 5: Figure S5: Dot-blot of cells transformed with scaffoldin-anchoring plasmids (cwa1, cwa2 and cwa3) or internal control (No-cwa) using a specific antibody against the CBM. As a positive control, the pure recombinant scaffoldin Scaf•AT was also applied to the blot. (TIFF 166 KB)

Additional file 6: Figure S6: Effect of MRS components on the ability of the pure recombinant Scaf•AT to bind to a cellulose-coated slide. Lanes 1 to 12 are numbered as follows: MRS, Tween 80, dextrose, yeast extract, beef extract, proteose peptone, TBS (as negative control), K_2_PO_4_, MnSO_4_, MgSO_4_, sodium acetate and ammonium citrate respectively. (TIFF 218 KB)

Additional file 7: Figure S7: Effect of Tween 80 or proteose peptone removal from MRS on *L. plantarum* growth curve. (TIFF 2 MB)

Additional file 8: Figure S8: Western blot analysis of culture supernatant fluids from transformed lactobacilli. A. Lanes 1 to 3: endoglucanase 6A-*t* expressed with the Lp1, Lp2 and No-Lp plasmids, respectively. B. Lanes 1 and 2: xylanase 11A-*a* expressed with the Lp1 and No-Lp plasmids, respectively. The calculated masses of secreted 6A-*t* and 11A-*a* are 40 kDa and 42.1 kDa, respectively. (TIFF 610 KB)

Additional file 9: Figure S9: Enzymatic activity in supernatant fluids of co-cultures producing the cellulase and the xylanase internally (No-Lp) either with the anchored scaffoldin (first group of bars (cwa2) or with the scaffoldin expressed internally (No-cwa) on hypochlorite pretreated wheat straw (grey bars) as opposed to the cell-wall assembled designer cellulosome (orange bar). (TIFF 2 MB)

## Abbreviations

BSA: Bovine serum albumin
CBM: Carbohydrate-binding module
CBP: Consolidated bioprocessing
CFP: Cyan fluorescent protein
cwa: Cell-wall anchor
ddw: double-distilled water
DIC: differential interference contrast
DM: dichroic mirror
DNS: Dinitrosalicylic acid
ECL: Enhanced chemiluminescence
EDTA: Ethylenediaminetetraacetic acid
HRP: Horseradish peroxidase
Lp: Leader peptide
PASC: Phosphoric acid swollen cellulose
SE: secondary electron
TBS: Tris-buffered saline
TMB: 3,3’,5,5’-Tetramethylbenzidine
YFP: Venus yellow fluorescent protein.

## References

[1] Cantarel BL, Coutinho PM, Rancurel C, Bernard T, Lombard V, Henrissat B (2009). The Carbohydrate-Active EnZymes database (CAZy): an expert resource for Glycogenomics. Nucleic Acids Res.

[2] Himmel M, Xu Q, Luo Y, Ding S, Lamed R, Bayer E (2010). Microbial enzyme systems for biomass conversion: Emerging paradigms. Biofuels.

[3] Wilson DB (2004). Studies of *Thermobifida fusca* plant cell wall degrading enzymes. Chem Rec.

[4] Ezer A, Matalon E, Jindou S, Borovok I, Atamna N, Yu Z, Morrison M, Bayer EA, Lamed R (2008). Cell surface enzyme attachment is mediated by family 37 carbohydrate-binding modules, unique to *Ruminococcus albus*. J Bacteriol.

[5] Elkins JG, Raman B, Keller M (2010). Engineered microbial systems for enhanced conversion of lignocellulosic biomass. Curr Opin Biotechnol.

[6] Huang GL, Anderson TD, Clubb RT (2014). Engineering microbial surfaces to degrade lignocellulosic biomass. Bioengineered.

[7] Aho S, Arffman A, Korhola M (1996). *Saccharomyces cerevisiae* mutants selected for increased production of *Trichoderma reesei* cellulases. Appl Microbiol Biotechnol.

[8] Ilmen M, den Haan R, Brevnova E, McBride J, Wiswall E, Froehlich A, Koivula A, Voutilainen SP, Siika-Aho M, la Grange DC, Thorngren N, Ahlgren S, Mellon M, Deleault K, Rajgarhia V, van Zyl WH, Penttila M (2011). High level secretion of cellobiohydrolases by *Saccharomyces cerevisiae*. Biotechnol Biofuels.

[9] Gobius KS, Xue GP, Aylward JH, Dalrymple BP, Swadling YJ, McSweeney CS, Krause DO (2002). Transformation and expression of an anaerobic fungal xylanase in several strains of the rumen bacterium *Butyrivibrio fibrisolvens*. J Appl Microbiol.

[10] Francisco JA, Stathopoulos C, Warren RAJ, Kilburn DG, Georgiou G (1993). Specific adhesion and hydrolysis of cellulose by intact *Escherichia coli* cells expressing surface-anchored cellulase or cellulose-binding domains. Bio/Technol.

[11] Kim JH, Park IS, Kim BG (2005). Development and characterization of membrane surface display system using molecular chaperon, prsA, of Bacillus subtilis. Biochem Biophys Res Commun.

[12] Kim YS, Jung HC, Pan JG (2000). Bacterial cell surface display of an enzyme library for selective screening of improved cellulase variants. Appl Environ Microbiol.

[13] Yeasmin S, Kim CH, Park HJ, Sheikh MI, Lee JY, Kim JW, Back KK, Kim SH (2011). Cell surface display of cellulase activity-free xylanase enzyme on *Saccharomyces Cerevisiae* EBY100. Appl Biochem Biotechnol.

[14] Matano Y, Hasunuma T, Kondo A (2013). Cell recycle batch fermentation of high-solid lignocellulose using a recombinant cellulase-displaying yeast strain for high yield ethanol production in consolidated bioprocessing. Bioresour Technol.

[15] Matano Y, Hasunuma T, Kondo A (2013). Simultaneous improvement of saccharification and ethanol production from crystalline cellulose by alleviation of irreversible adsorption of cellulase with a cell surface-engineered yeast strain. Appl Microbiol Biotechnol.

[16] Yanase S, Yamada R, Kaneko S, Noda H, Hasunuma T, Tanaka T, Ogino C, Fukuda H, Kondo A (2010). Ethanol production from cellulosic materials using cellulase-expressing yeast. Biotechnol J.

[17] Fujita Y, Ito J, Ueda M, Fukuda H, Kondo A (2004). Synergistic saccharification, and direct fermentation to ethanol, of amorphous cellulose by use of an engineered yeast strain codisplaying three types of cellulolytic enzyme. Appl Environ Microbiol.

[18] Lilly M, Fierobe HP, van Zyl WH, Volschenk H (2009). Heterologous expression of a *Clostridium* minicellulosome in *Saccharomyces cerevisiae*. FEMS Yeast Res.

[19] Tsai SL, Oh J, Singh S, Chen R, Chen W (2009). Functional assembly of minicellulosomes on the *Saccharomyces cerevisiae* cell surface for cellulose hydrolysis and ethanol production. Appl Environ Microbiol.

[20] Wen F, Sun J, Zhao H (2010). Yeast surface display of trifunctional minicellulosomes for simultaneous saccharification and fermentation of cellulose to ethanol. Appl Environ Microbiol.

[21] Tsai SL, Dasilva NA, Chen W (2013). Functional Display of Complex Cellulosomes on the Yeast Surface via Adaptive Assembly. ACS Synthetic Biol.

[22] Fan LH, Zhang ZJ, Yu XY, Xue YX, Tan TW (2012). Self-surface assembly of cellulosomes with two miniscaffoldins on *Saccharomyces cerevisiae* for cellulosic ethanol production. Proc Nat Acad Sci USA.

[23] Kim S, Baek SH, Lee K, Hahn JS (2013). Cellulosic ethanol production using a yeast consortium displaying a minicellulosome and beta-glucosidase. Microbial Cell Factories.

[24] Wieczorek AS, Martin VJ (2010). Engineering the cell surface display of cohesins for assembly of cellulosome-inspired enzyme complexes on *Lactococcus lactis*. Microbial Cell Factories.

[25] Anderson TD, Robson SA, Jiang XW, Malmirchegini GR, Fierobe HP, Lazazzera BA, Clubb RT (2011). Assembly of minicellulosomes on the surface of *Bacillus subtilis*. Appl Environ Microbiol.

[26] Kovacs K, Willson BJ, Schwarz K, Heap JT, Jackson A, Bolam DN, Winzer K, Minton NP (2013). Secretion and assembly of functional mini-cellulosomes from synthetic chromosomal operons in *Clostridium acetobutylicum* ATCC 824. Biotechnol Biofuels.

[27] Gaspar P, Carvalho AL, Vinga S, Santos H, Neves AR (2013). From physiology to systems metabolic engineering for the production of biochemicals by lactic acid bacteria. Biotechnol Adv.

[28] Alegria EG, Lopez I, Ruiz JI, Saenz J, Fernandez E, Zarazaga M, Dizy M, Torres C, Ruiz Larrea F (2004). High tolerance of wild *Lactobacillus plantarum* and *Oenococcus oeni* strains to lyophilisation and stress environmental conditions of acid pH and ethanol. FEMS Microbiol Lett.

[29] Wood BE, Beall DS, Ingram LO (1997). Production of recombinant bacterial endoglucanase as a co-product with ethanol during fermentation using derivatives of *Escherichia coli* KO11. Biotechnol Bioeng.

[30] Morais S, Shterzer N, Rozman Grinberg I, Mathiesen G, Eijsink VG, Axelsson L, Lamed R, Bayer EA, Mizrahi I (2013). Establishment of a simple *Lactobacillus plantarum* cell consortium for cellulase-xylanase synergistic interactions. Appl Environ Microbiol.

[31] Agapakis CM, Boyle PM, Silver PA (2012). Natural strategies for the spatial optimization of metabolism in synthetic biology. Nat Chem Biol.

[32] Arai T, Matsuoka S, Cho HY, Yukawa H, Inui M, Wong SL, Doi RH (2007). Synthesis of *Clostridium cellulovorans* minicellulosomes by intercellular complementation. Proc Nat Acad Sci USA.

[33] Fierobe H-P, Bayer EA, Tardif C, Czjzek M, Mechaly A, Belaich A, Lamed R, Shoham Y, Belaich J-P (2002). Degradation of cellulose substrates by cellulosome chimeras: Substrate targeting versus proximity of enzyme components. J Biol Chem.

[34] Fierobe H-P, Mechaly A, Tardif C, Belaich A, Lamed R, Shoham Y, Belaich J-P, Bayer EA (2001). Design and production of active cellulosome chimeras: Selective incorporation of dockerin-containing enzymes into defined functional complexes. J Biol Chem.

[35] Fierobe H-P, Mingardon F, Mechaly A, Belaich A, Rincon MT, Lamed R, Tardif C, Belaich J-P, Bayer EA (2005). Action of designer cellulosomes on homogeneous versus complex substrates: Controlled incorporation of three distinct enzymes into a defined tri-functional scaffoldin. J Biol Chem.

[36] Moraïs S, Barak Y, Caspi J, Hadar Y, Lamed R, Shoham Y, Wilson DB, Bayer EA (2010). Contribution of a xylan-binding module to the degradation of a complex cellulosic substrate by designer cellulosomes. Appl Environ Microbiol.

[37] Moraïs S, Barak Y, Caspi J, Hadar Y, Lamed R, Shoham Y, Wilson DB, Bayer EA (2010). Cellulase-xylanase synergy in designer cellulosomes for enhanced degradation of a complex cellulosic substrate. mBio.

[38] Morais S, Barak Y, Hadar Y, Wilson DB, Shoham Y, Lamed R, Bayer EA (2011). Assembly of xylanases into designer cellulosomes promotes efficient hydrolysis of the xylan component of a natural recalcitrant cellulosic substrate. mBio.

[39] Morais S, Morag E, Barak Y, Goldman D, Hadar Y, Lamed R, Shoham Y, Wilson DB, Bayer EA (2013). Deconstruction of lignocellulose into soluble sugars by native and designer cellulosomes. mBio.

[40] Barak Y, Handelsman T, Nakar D, Mechaly A, Lamed R, Shoham Y, Bayer EA (2005). Matching fusion-protein systems for affinity analysis of two interacting families of proteins: the cohesin-dockerin interaction. J Mol Recogn.

[41] Fredriksen L, Mathiesen G, Sioud M, Eijsink VG (2010). Cell wall anchoring of the 37-kilodalton oncofetal antigen by *Lactobacillus plantarum* for mucosal cancer vaccine delivery. Appl Environ Microbiol.

[42] Caspi J, Irwin D, Lamed R, Shoham Y, Fierobe H-P, Wilson DB, Bayer EA (2006). *Thermobifida fusca* family-6 cellulases as potential designer cellulosome components. Biocatalys Biotransform.

[43] Caspi J, Barak Y, Haimovitz R, Gilary H, Irwin DC, Lamed R, Wilson DB, Bayer EA (2010). *Thermobifida fusca* exoglucanase Cel6B is incompatible with the cellulosomal mode in contrast to endoglucanase Cel6A. Syst Syn Biol.

[44] Mathiesen G, Sveen A, Brurberg MB, Fredriksen L, Axelsson L, Eijsink VGH (2009). Genome-wide analysis of signal peptide functionality in *Lactobacillus plantarum* WCFS1. BMC Genomics.

[45] Bansal P, Hall M, Realff MJ, Lee JH, Bommarius AS (2009). Modeling cellulase kinetics on lignocellulosic substrates. Biotechnol Adv.

[46] Kostylev M, Wilson D (2013). Two-parameter kinetic model based on a time-dependent activity coefficient accurately describes enzymatic cellulose digestion. Biochemistry.

[47] Okano K, Zhang Q, Yoshida S, Tanaka T, Ogino C, Fukuda H, Kondo A (2010). D-lactic acid production from cellooligosaccharides and beta-glucan using L-LDH gene-deficient and endoglucanase-secreting *Lactobacillus plantarum*. Appl Microbiol Biotechnol.

[48] Probst M, Fritschi A, Wagner A, Insam H (2013). Biowaste: a *Lactobacillus* habitat and lactic acid fermentation substrate. Bioresour Technol.

[49] Hasunuma T, Okazaki F, Okai N, Hara KY, Ishii J, Kondo A (2013). A review of enzymes and microbes for lignocellulosic biorefinery and the possibility of their application to consolidated bioprocessing technology. Bioresour Technol.

[50] Mizrahi I, Rosenberg EDF, Lory S, Stackebrandt E, Thompson F (2013). Rumen symbioses. The Prokaryotes.

[51] Bayer EA, Kenig R, Lamed R (1983). Adherence of *Clostridium thermocellum* to cellulose. J Bacteriol.

[52] Francisco JA, Stathopoulos C, Warren RA, Kilburn DG, Georgiou G (1993). Specific adhesion and hydrolysis of cellulose by intact *Escherichia coli* expressing surface anchored cellulase or cellulose binding domains. Biotechnol (N Y).

[53] Han Z, Zhang B, Wang YE, Zuo YY, Su WW (2012). Self-assembled amyloid-like oligomeric-cohesin Scaffoldin for augmented protein display on the saccharomyces cerevisiae cell surface. Appl Environ Microbiol.

[54] Le Costaouec T, Pakarinen A, Varnai A, Puranen T, Viikari L (2013). The role of carbohydrate binding module (CBM) at high substrate consistency: comparison of *Trichoderma reesei* and *Thermoascus aurantiacus* Cel7A (CBHI) and Cel5A (EGII). Bioresour Technol.

[55] Ghangas GS, Wilson DB (1988). Cloning of the *Thermomonospora fusca* Endoglucanase E2 Gene in *Streptomyces lividans*: affinity Purification and Functional Domains of the Cloned Gene Product. Appl Environ Microbiol.

[56] Sorvig E, Mathiesen G, Naterstad K, Eijsink VGH, Axelsson L (2005). High-level, inducible gene expression in *Lactobacillus sakei* and *Lactobacillus plantarum* using versatile expression vectors. Microbiology.

[57] Gasteiger EHC, Gattiker A, Duvaud S, Wilkins MR, Appel RD, Bairoch A, Walker JM (2005). Protein Identification and Analysis Tools on the ExPASy Server. The Proteomics Protocols Handbook.

[58] Aukrust T, Blom H (1992). Transformation of *Lactobacillus* strains used in meat and vegetable fermentations. Food Res Int.

[59] Eijsink VG, Brurberg MB, Middelhoven PH, Nes IF (1996). Induction of bacteriocin production in Lactobacillus sake by a secreted peptide. J Bacteriol.

[60] Miller GL (1959). Use of dinitrosalicylic acid reagent for determination of reducing sugar. Anal Biochem.

[61] Ghose TK (1987). Measurments of cellulase activity. Pure Appl Chem.

[62] Tabka MG, Herpoël-Gimbert I, Monod F, Asther M, Sigoillot JC (2006). Enzymatic saccharification of wheat straw for bioethanol production by a combined cellulase xylanase and feruloyl esterase treatment. Enzyme Microb Technol.

[63] Morais S, Morag E, Barak Y, Goldman D, Hadar Y, Lamed R, Shoham Y, Wilson DB, Bayer EA (2012). Deconstruction of lignocellulose into soluble sugars by native and designer cellulosomes. mBio.

