# Supplementary figures and images for: A combined cell-consortium approach for lignocellulose degradation by specialized Lactobacillus plantarum cells
Source: Biotechnol Biofuels. 2014 Jul 24;7:112. doi: 10.1186/1754-6834-7-112 (PMC4364503; doi:10.1186/1754-6834-7-112)

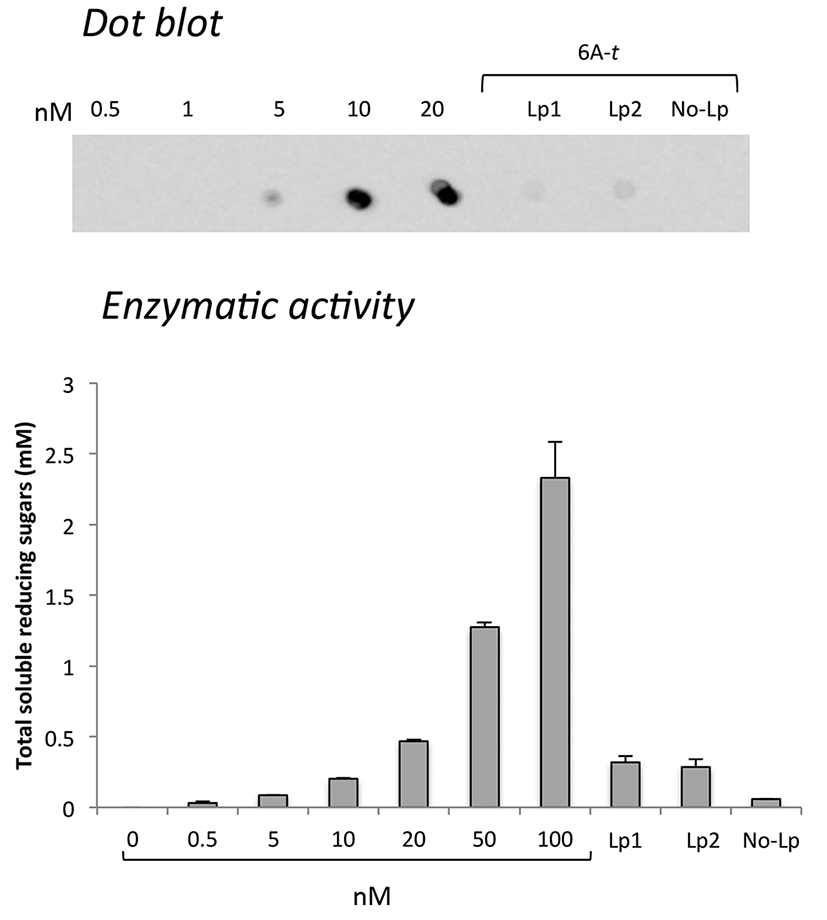

Supplement: Supplementary file 1 — Additional file 1: Figure S1: Quantification of secreted cellulase 6A-t by dot-blot and enzymatic activity using known concentrations of the recombinant protein produced in E. coli. (TIFF 783 KB) [file 13068_2014_508_MOESM1_ESM.tiff]

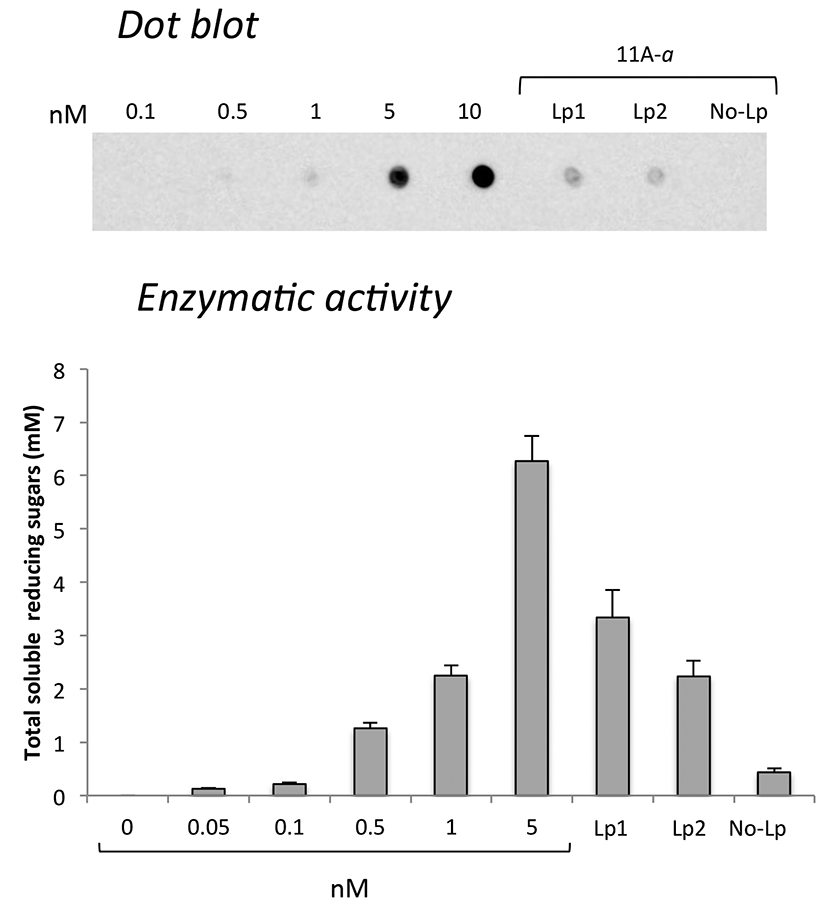

Supplement: Supplementary file 2 — Additional file 2: Figure S2: Quantification of secreted xylanase 11A-a by dot-blot and enzymatic activity using known concentrations of the recombinant protein produced in E. coli. (TIFF 754 KB) [file 13068_2014_508_MOESM2_ESM.tiff]

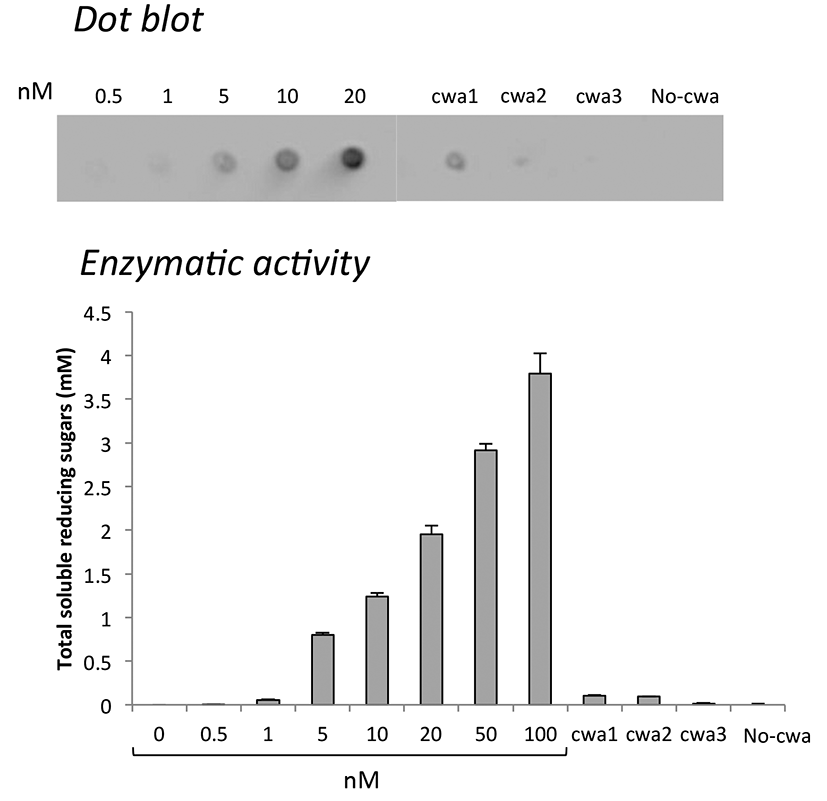

Supplement: Supplementary file 3 — Additional file 3: Figure S3: Quantification of anchored cellulase Cel6A by dot-blot and enzymatic activity using known concentrations of the recombinant protein produced in E. coli. (TIFF 669 KB) [file 13068_2014_508_MOESM3_ESM.tiff]

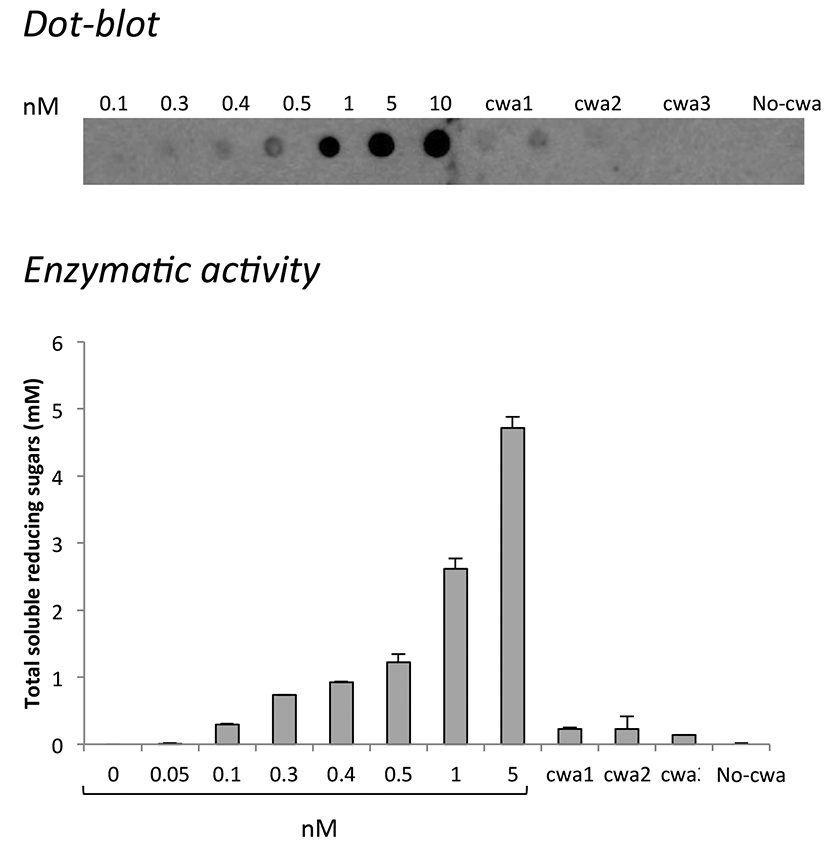

Supplement: Supplementary file 4 — Additional file 4: Figure S4: Quantification of anchored xylanase Xyn11A by dot-blot and enzymatic activity using known concentrations of the recombinant protein produced in E. coli. (TIFF 704 KB) [file 13068_2014_508_MOESM4_ESM.tiff]

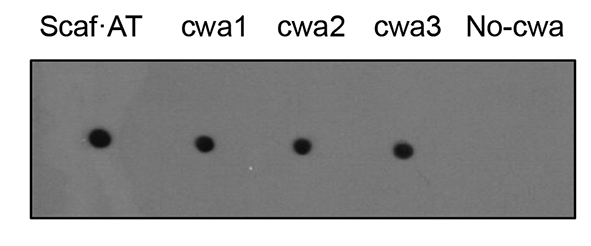

Supplement: Supplementary file 5 — Additional file 5: Figure S5: Dot-blot of cells transformed with scaffoldin-anchoring plasmids (cwa1, cwa2 and cwa3) or internal control (No-cwa) using a specific antibody against the CBM. As a positive control, the pure recombinant scaffoldin Scaf•AT was also applied to the blot. (TIFF 166 KB) [file 13068_2014_508_MOESM5_ESM.tiff]

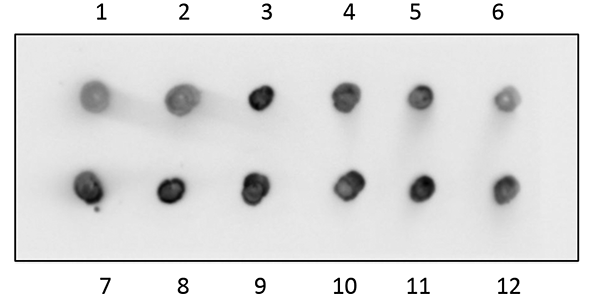

Supplement: Supplementary file 6 — Additional file 6: Figure S6: Effect of MRS components on the ability of the pure recombinant Scaf•AT to bind to a cellulose-coated slide. Lanes 1 to 12 are numbered as follows: MRS, Tween 80, dextrose, yeast extract, beef extract, proteose peptone, TBS (as negative control), K2PO4, MnSO4, MgSO4, sodium acetate and ammonium citrate respectively. (TIFF 218 KB) [file 13068_2014_508_MOESM6_ESM.tiff]

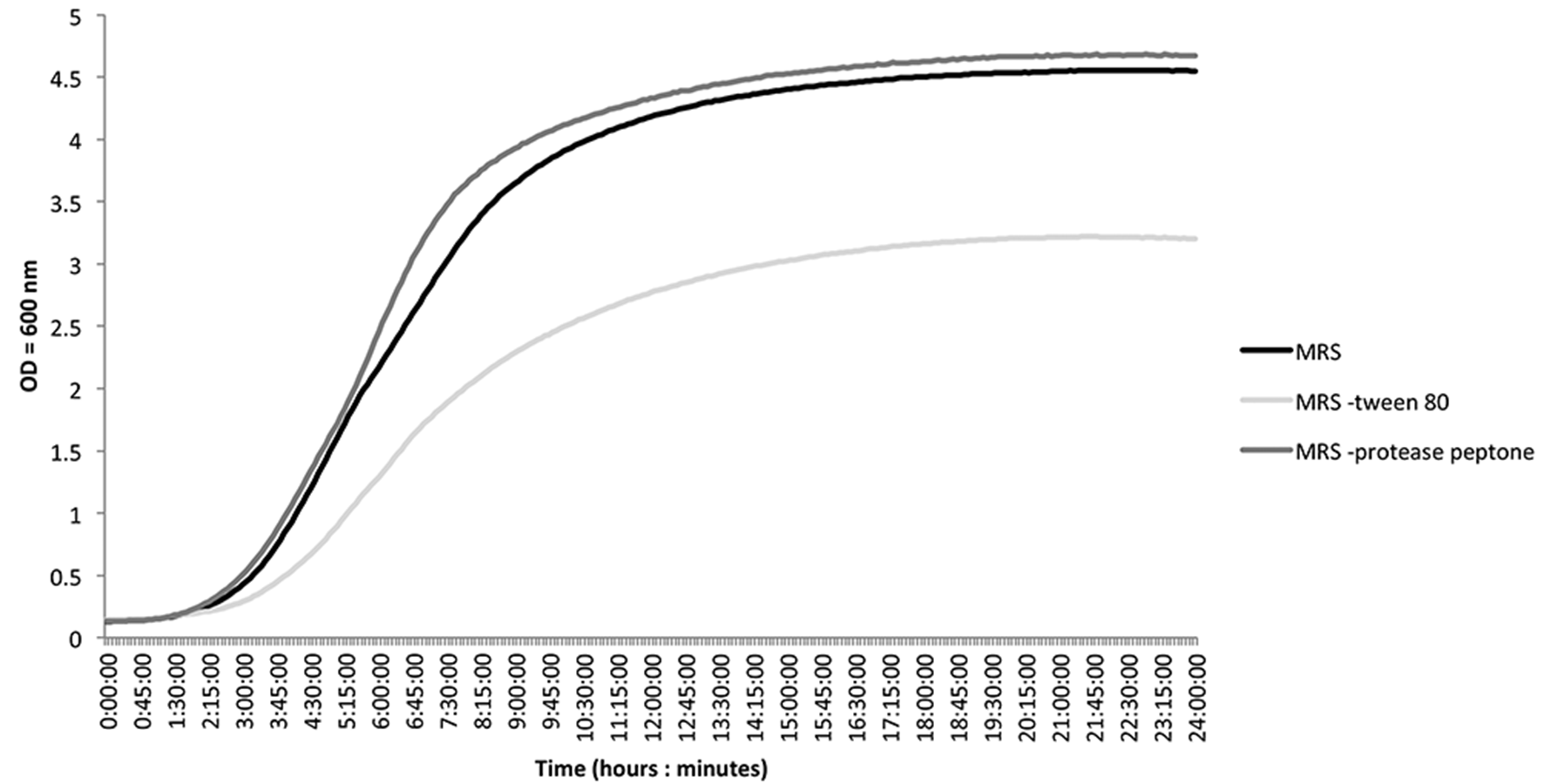

Supplement: Supplementary file 7 — Additional file 7: Figure S7: Effect of Tween 80 or proteose peptone removal from MRS on L. plantarum growth curve. (TIFF 2 MB) [file 13068_2014_508_MOESM7_ESM.tiff]

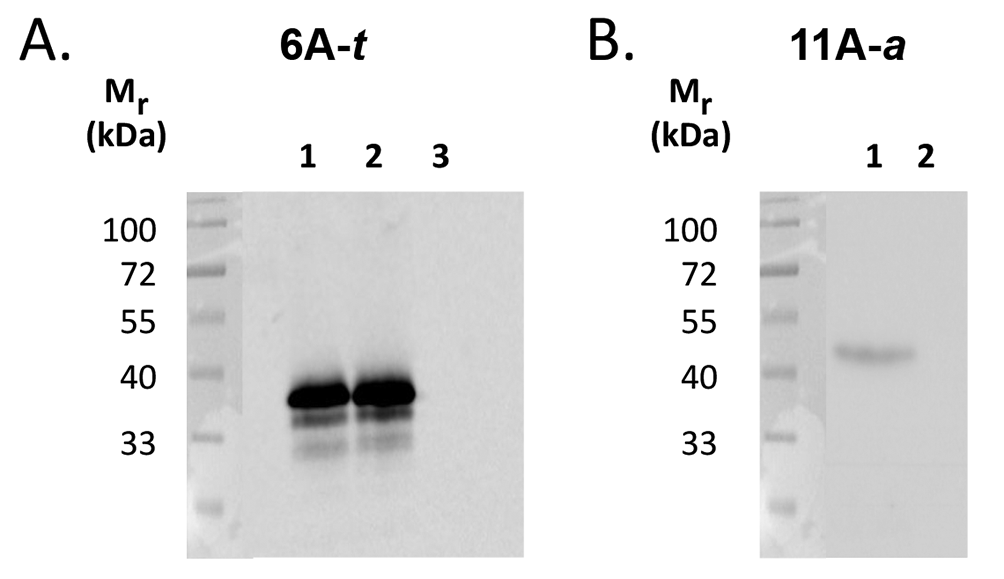

Supplement: Supplementary file 8 — Additional file 8: Figure S8: Western blot analysis of culture supernatant fluids from transformed lactobacilli. A. Lanes 1 to 3: endoglucanase 6A-t expressed with the Lp1, Lp2 and No-Lp plasmids, respectively. B. Lanes 1 and 2: xylanase 11A-a expressed with the Lp1 and No-Lp plasmids, respectively. The calculated masses of secreted 6A-t and 11A-a are 40 kDa and 42.1 kDa, respectively. (TIFF 610 KB) [file 13068_2014_508_MOESM8_ESM.tiff]

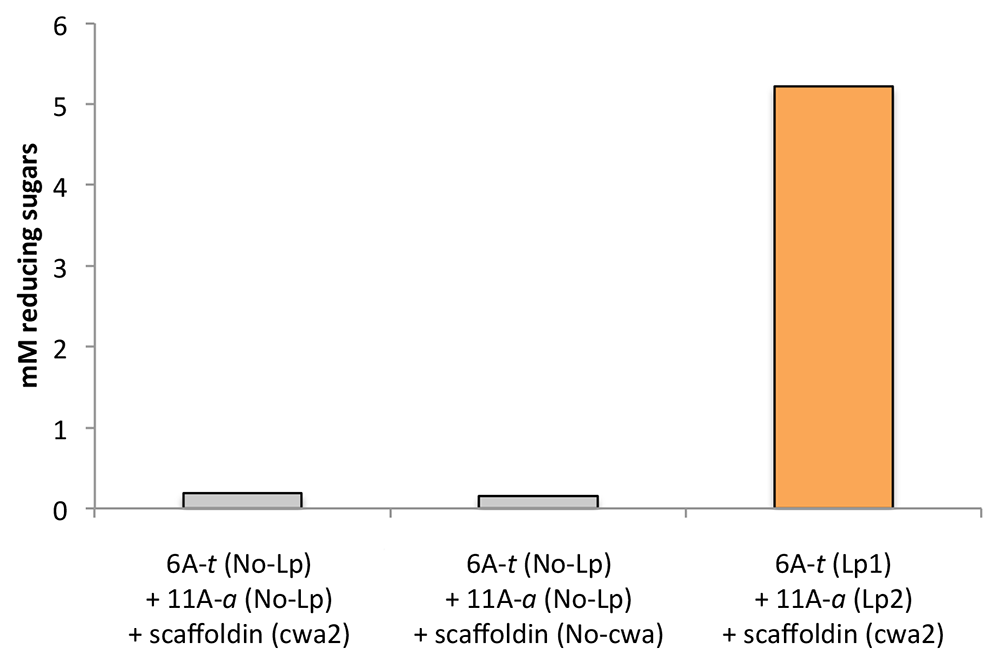

Supplement: Supplementary file 9 — Additional file 9: Figure S9: Enzymatic activity in supernatant fluids of co-cultures producing the cellulase and the xylanase internally (No-Lp) either with the anchored scaffoldin (first group of bars (cwa2) or with the scaffoldin expressed internally (No-cwa) on hypochlorite pretreated wheat straw (grey bars) as opposed to the cell-wall assembled designer cellulosome (orange bar). (TIFF 2 MB) [file 13068_2014_508_MOESM9_ESM.tiff]
